# Resveratrol Derivatives as Potential Treatments for Alzheimer’s and Parkinson’s Disease

**DOI:** 10.3389/fnagi.2020.00103

**Published:** 2020-04-17

**Authors:** Bruno Dutra Arbo, Corinne André-Miral, Raif Gregorio Nasre-Nasser, Lúcia Emanueli Schimith, Michele Goulart Santos, Dennis Costa-Silva, Ana Luiza Muccillo-Baisch, Mariana Appel Hort

**Affiliations:** ^1^Instituto de Ciências Básicas da Saúde, Universidade Federal do Rio Grande do Sul (UFRGS), Porto Alegre, Brazil; ^2^Université de Nantes, CNRS, Unité de Fonctionnalité et Ingénierie des Protéines (UFIP), UMR 6286, Nantes, France; ^3^Instituto de Ciências Biológicas, Universidade Federal do Rio Grande (FURG), Rio Grande, Brazil

**Keywords:** neurodegenerative diseases, neurodegeneration, neuroprotection, polyphenols, stilbene, aging

## Abstract

Neurodegenerative diseases are characterized by the progressive loss of neurons in different regions of the nervous system. Alzheimer’s disease (AD) and Parkinson’s disease (PD) are the two most prevalent neurodegenerative diseases, and the symptoms associated with these pathologies are closely related to the regions that are most affected by the process of neurodegeneration. Despite their high prevalence, currently, there is no cure or disease-modifying drugs for the treatment of these conditions. In the last decades, due to the need for the development of new treatments for neurodegenerative diseases, several authors have investigated the neuroprotective actions of naturally occurring molecules, such as resveratrol. Resveratrol is a stilbene found in several plants, including grapes, blueberries, raspberries, and peanuts. Studies have shown that resveratrol presents neuroprotective actions in experimental models of AD and PD, however, its clinical application is limited due to its rapid metabolism and low bioavailability. In this context, studies have proposed that structural changes in the resveratrol molecule, including glycosylation, alkylation, halogenation, hydroxylation, methylation, and prenylation could lead to the development of derivatives with enhanced bioavailability and pharmacological activity. Therefore, this review article aims to discuss how resveratrol derivatives could represent viable molecules in the search for new drugs for the treatment of AD and PD.

## Introduction

Neurodegenerative diseases encompass a set of pathologies characterized by progressive neuronal death in specific encephalic regions, which produce different clinical manifestations according to the location of vulnerable neurons (Fu et al., 2018). This selective neuronal vulnerability has been associated to the aggregation of misfolded proteins that include: β-amyloid (Aβ) and Tau in Alzheimer’s disease (AD); α-synuclein (α-syn) in Parkinson’s disease (PD), dementia with Lewy bodies and multiple system atrophy; huntingtin in Huntington’s disease; TDP-43 in frontotemporal lobar degeneration and amyotrophic lateral sclerosis; among others (Dugger and Dickson, 2017; Fu et al., 2018). Among these, AD and PD are considered the most prevalent neurodegenerative diseases (Mayeux and Stern, 2012; Erkkinen et al., 2018), so they will be the focus of this review article.

In 2016, global estimates indicated that 43.8 million people were living with dementia, of which approximately 60% were due to AD, varying depending on the country and study methodology (Erkkinen et al., 2018; Nichols et al., 2019). In this sense, there was an increase of twice the number of cases of patients with dementia in 20 years, being more frequent in women than in men with a ratio of 1.2:1.0 (Prince et al., 2015; Nichols et al., 2019). On the other hand, it is estimated that in 2016 about six million people had PD worldwide, 2.4 times higher than the estimated prevalence for 1990; with a male-to-female ratio of 1.4–2.0:1.0 (Lee and Gilbert, 2016; Ray Dorsey et al., 2018). Both genetic predisposition and environmental factors have been involved in the clinical manifestation of AD and PD, with age being considered a major risk factor for both diseases (Lee and Gilbert, 2016; Alzheimer’s Association, 2019).

A progressive loss of functions with the consequent disability and premature mortality of patients with AD and PD have important implications for the quality of life of patients and family members. In this regard, a worldwide estimate of the disability-adjusted life-years (the sum of years lived with disability and years of life lost) evidenced that all dementias, including AD, caused 6.4 million, meanwhile, PD produced 3.2 million in 2016 (Ray Dorsey et al., 2018; Nichols et al., 2019). In the economic field, the costs associated with the treatment and care of patients with dementia associated with AD or other diseases is estimated to reach US $290 billion in 2019 in the USA (Alzheimer’s Association, 2019). On the other hand, the direct and indirect cost associated with PD, including treatment, social security payments and lost revenue, is estimated at US $52 billion per year only in the USA (Parkinson’s Foundation, 2019). Finally, it is important to mention that these data do not include the millions of dollars invested each year in the search for new treatments for these diseases (Cummings et al., 2018; Marshall and Willett, 2018).

Despite the high impact of AD and PD in social and economic spheres, currently, there is no cure or disease-modifying treatment for most of the cases (Gitler et al., 2017). Nowadays, the treatment for these diseases is based on pharmacological and non-pharmacological strategies aimed to improve the syndromic picture of these pathologies. Thereby, acetylcholinesterase inhibitors (e.g., donepezil, galantamine, and rivastigmine) and N-methyl-D-aspartate (NMDA) receptor antagonists (e.g., memantine) are used for AD treatment. On the other hand, drugs that increase the synaptic availability of dopamine, such as the precursor of this neurotransmitter (levodopa) and inhibitors of enzymes involved in dopamine metabolism (e.g., carbidopa, entacapone, rasagiline), as well as agonists of dopamine receptors (e.g., pramipexole, ropinirole), are prescribed for patients with PD (Oertel and Schulz, 2016; Van Bulck et al., 2019).

The development of new treatments to combat neurodegenerative diseases requires a better understanding of the underlying pathophysiological processes, elucidating how genetic alterations interact with environmental factors to produce neurodegeneration. Thus, alterations of proteostasis, mitochondrial function, autophagy, redox status, metal and calcium metabolism, as well as neuroinflammation and neurogenesis have become potential therapeutic drug targets that are expected to modify the progression of these illnesses (Van Bulck et al., 2019; Elkouzi et al., 2019). However, obtaining an effective and safe drug for these diseases entails great difficulty considering that not all the molecules evaluated in preclinical studies are viable during the development of clinical trials; for example, between 2002 and 2012, there were 244 drugs registered in the National Institutes of Health (NIH) for clinical studies, and only one received permission to be used in patients with AD (Alzheimer’s Association, 2018). Examples of phase 3 clinical trials that have failed include those involving the anti-Aβ antibodies bapineuzumab (Salloway et al., 2014; Vandenberghe et al., 2016) and solanezumab (Honig et al., 2018), and the inhibitors of β-secretase and γ-secretase verubecestat (Egan et al., 2018) and semagacestat (Doody et al., 2013), respectively, which inhibit the enzymes involved in Aβ production. At the beginning of 2019, 132 agents were being tested in clinical trials for AD, including 28 agents being evaluated in 42 phase 3 clinical trials (Cummings et al., 2019), and there is a lot of expectation regarding the revelation of the results of these studies. The panorama for the development of new drugs for the treatment of PD is not very different, and among some unsuccessful attempts are CEP-1347, a mixed lineage kinases inhibitor (Shoulson et al., 2007), some adenosine A_2A_ receptor antagonists such as preladenant, vipadenant and tozadenant (Pinna, 2014; National Library of Medicine, 2019), isradipine, a dihydropyridine calcium channel antagonist (Lee et al., 2017; Royal Pharmaceutical Society, 2019), and inosine, that is considered to act as an antioxidant and anti-inflammatory (National Library of Medicine, 2018; National Institute of Health, 2019).

Several studies made in the last years have focused on the research of the therapeutic potential of a large number of natural compounds for neurodegenerative diseases, especially those derived from plant extracts (Wąsik and Antkiewicz-Michaluk, 2017). Throughout history, ancestral and traditional wisdom about the medicinal use of some plants has been the base for the study of the mechanism of action and safety of countless extracts and compounds isolated from plants (Anton et al., 2008; More et al., 2013). The pharmacological attractiveness of the use of natural compounds revolves around their reduced side effects and their multiple target mechanism of action, which is particularly useful for the prevention or treatment of multifactorial diseases such as those that produce neurodegeneration (Bagli et al., 2016; Pohl and Kong Thoo Lin, 2018). Within the vast diversity of natural compounds that are under study for their neuroprotective potential, are the polyphenolic compounds, including resveratrol (RV), curcumin, capsaicin and epigallocatechin gallate (Zhang et al., 2017; Habtemariam, 2019). With this background, the aim of the current review is to discuss the neuroprotective effects of RV and its derivatives in experimental models of AD and PD, addressing if these substances could lead to the development of new treatments for neurodegenerative diseases.

## Resveratrol

**Figure 1:**
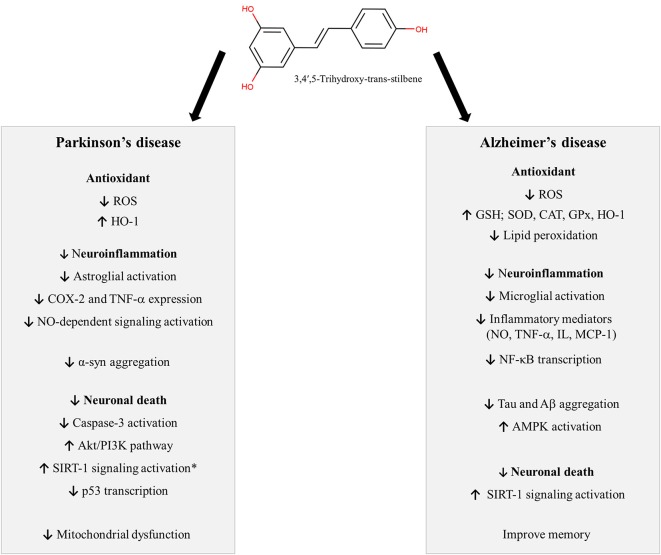
Neuroprotective mechanisms described for Resveratrol. *Some studies did not evidence any involvement of SIRT-1 in the neuroprotective actions of RV against MPP+. Symbols: ↓: decrease; ↑: increase. Abbreviations: α-syn: alfa-synuclein; Aβ: amyloid-beta, Akt: protein kinase B, AMPK: AMP-activated protein kinase, CAT: catalase, COX-2: cyclooxygenase-2, GPx: glutathione peroxidase, GSH: glutathione, HO-1: heme oxygenase 1, IL: interleukins, MCP-1: chemokine monocyte chemotactic protein-1, NF-κB: nuclear factor kappa B, NO: nitric oxide, p53: tumor protein p53, PI3K: phosphatidylinosi-tol 3-kinase, ROS: reactive oxygen species, SIRT-1: anti-aging factor sirtuin-1, SOD: superoxide dismutase, TNF-α: tumor necrosis factor-α.

Resveratrol (3,4′,5-trihydroxy-*trans*-stilbene) is a stilbene, a subclass of phenolic compounds, found in several plants, including grapes, blueberries, raspberries, and peanuts. The stilbene structure is composed of 14 carbon basic skeleton and two phenyl groups linked by an ethene double bond (Figure 1; He and Yan, 2013; Tsai et al., 2017). Experimental studies have shown that stilbenoids, such as RV, present antioxidant and anti-inflammatory activity, leading to neuroprotection (Figure 1). In this section, we will discuss the neuroprotective effects of RV in different experimental models (preclinical studies), exploring its mechanism of action. Also, clinical studies in AD and PD and limitations of RV use will be reported.

### Preclinical Studies

*In vitro* or animal models can mimic some features from AD and PD pathophysiology, thus providing information about potential therapeutic targets and new drugs for the treatment of these conditions. Both AD and PD are associated with inflammation and oxidative damage. Therefore, antioxidant and anti-inflammatory agents may be useful tools for the development of new treatments against these diseases. In this context, several studies have demonstrated that RV presents antioxidant and anti-inflammatory actions. Zhang et al. (2010) showed that RV protected dopaminergic neurons against lipopolysaccharide-induced neurotoxicity through the inhibition of microglial activation and nuclear factor kappa B (NF-κB) signaling. In agreement, Chen et al. (2017) verified that RV decreased the mitochondrial oxidative stress and apoptosis in the hippocampus of mice treated with LPS. Furthermore, RV and one of its metabolites protect HT22 neuronal cells against glutamate-induced neuronal oxidative stress through the induction of nuclear factor erythroid 2-related factor (Nrf2)-dependent heme oxygenase 1 (HO-1) expression (Kim et al., 2012; Son et al., 2013). These data are supported by other studies, as reviewed by Truong et al. (2018), which show that the levels of key antioxidant transcription factors such as Nrf2, HO-1, and glutathione S-transferase (GST) are increased by RV. Therefore, since RV presents antioxidant and anti-inflammatory actions, several studies have investigated its neuroprotective actions in experimental models of AD and PD.

#### Neuroprotective Effects of Resveratrol in Alzheimer’s Disease

The neuroprotective effects of RV have been investigated in several *in vitro* and *in vivo* experimental models of AD (Feng et al., 2009, 2013; Karuppagounder et al., 2009; Porquet et al., 2014; Freyssin et al., 2020; Rao et al., 2020). RV can modify the underlying pathology of AD by several mechanisms which may slow the onset and progression of the disease (Ahmed et al., 2017; Sawda et al., 2017). Among the mechanisms of action of RV in AD we can highlight its antioxidant action, reduction of neuroinflammation, inhibition of tauopathy and Aβ-plaque formation, therefore inhibiting neuronal death and improving memory (Ahmed et al., 2017). The wide variety of pharmacological targets of RV may be an advantage in its use as a neuroprotective agent (Andrade et al., 2019).

Oxidative stress plays an essential role in the pathogenesis of AD. Increased production of reactive oxygen species (ROS) associated with mitochondrial dysfunction, altered metal homeostasis and decreased antioxidant defenses affect synaptic activity and cause neuron damage in AD. In this context, antioxidant compounds, like RV may be useful for the prevention and treatment of the disease (Chen and Zhong, 2014; Tönnies and Trushina, 2017). Several studies suggest that RV protects against Aβ-induced oxidative damage in different experimental AD models *in vitro* (Conte et al., 2003; Jang and Surh, 2003; Chiang et al., 2018; Wang et al., 2018b) and *in vivo* (Karuppagounder et al., 2009; Kong et al., 2019). RV can exert protection against neuronal oxidative damage in different ways. It can increase the intracellular antioxidant levels, such as glutathione (Sharma and Gupta, 2002; Savaskan et al., 2003; Kwon et al., 2010) and antioxidant enzymes, such as superoxide dismutase (SOD), catalase (CAT), glutathione peroxidase (GPx) and HO-1 (Chiang et al., 2018; Lin et al., 2018; Zhao et al., 2018; Kong et al., 2019), and decrease lipid peroxidation (Sharma and Gupta, 2002; Kong et al., 2019). Moreover, RV prevents the disruption of mitochondrial membrane potential, reducing ROS production in brain tissue (Kwon et al., 2010). In lymphoblastoid cell lines (LCLs) from AD patients, RV increased the expression of genes encoding known antioxidants defenses (CAT, copper chaperone for SOD 1, GST zeta 1) and anti-aging factor sirtuin-1 (SIRT-1; Cosín-Tomàs et al., 2019). These results are corroborated by Zhang et al. (2019), which showed that RV improves learning and memory in rats submitted to a vascular dementia model, probably due to its antioxidant actions, represented by increased protein expression of SOD and a decreased hippocampal content of malondialdehyde (MDA).

Another well-explored mechanism of RV is its ability to prevent inflammatory response associated with AD. In AD, the Aβ deposition is known to activate glial cells, including both astrocytes and microglia, leading to neuroinflammation, which is often associated with neuronal death (Kinney et al., 2018). Moreover, evidence suggests that neuroinflammation induced by reactive microglia reduced Aβ clearance causing synaptic dysfunction and memory impairment (Wang W. Y. et al., 2015). In astrocytes, RV inhibits LPS induced production of inflammatory mediators, including nitric oxide (NO); tumor necrosis factor-α (TNF-α), interleukins (IL-1β, IL-6, IL-12p40, IL-23), chemokine monocyte chemotactic protein-1 (MCP-1) and C-reactive protein (CRP; Wight et al., 2012). Feng and Zhang (2019) investigated the effects of RV in a model of inflammatory injury induced by Aβ in murine microglial cells (BV-2 cells). RV significantly inhibited Aβ-induced proliferation and activation of BV-2 cells, as well as the release of the proinflammatory cytokines IL-6 and TNF-α. Several studies show that the anti-neuroinflammatory effect of RV is related to the inhibition of NF-κB. In rat astrocytes and microglial cell lines, RV reduces the inflammation induced by Aβ1–42 through the inhibition of the NF-κB signal pathway (Zhao et al., 2018). In rodents, a reduction of NK-κB activation was observed after RV treatment both in ovariectomized D-galactose rats exposed to Aβ1–42 (Zhao et al., 2015) and in transgenic mice expressing Aβ/presenilin-1 (Solberg et al., 2014). Interestingly, some studies demonstrated that ε-viniferin, a naturally occurring RV dimer, is also protective in experimental models of AD. This compound improved the cognition of mice that received an intracerebroventricular injection of Aβ25–35 (Jeong et al., 2010), as well as decreased amyloid deposits, astrogliosis and microglial activation in APPswePS1dE9 mice (Caillaud et al., 2019).

The neuroprotective action of RV has also been associated with its ability to increase the activity of SIRT-1 (Ahmed et al., 2017), a nicotinamide adenosine dinucleotide (NAD)-dependent deacetylase that removes acetyl groups from various proteins (Donmez and Outeiro, 2013). SIRT-1 regulates a wide variety of biological functions, including embryonic development, cell differentiation and apoptosis (Borra et al., 2005; Wang et al., 2018a). In AD, the accumulation of Aβ decreases the levels of SIRT-1 in neurons (Donmez and Outeiro, 2013; Jęśko et al., 2017; Xu et al., 2018). In this line, some studies reported that the preventive action of RV is probably related to the modulation of SIRT-1 expression and activity in neurons (Lagouge et al., 2006; Feng et al., 2013). These effects may play an important role in protecting neurons from oxidative damage, prevent Aβ toxicity and reduce inflammatory cytokines production by microglia, contributing to improving memory function (Gomes et al., 2018).

Another key target of AD is related to the microtubule-associated protein Tau, which forms insoluble filaments that accumulate as neurofibrillary tangles in AD and related tauopathies (Iqbal et al., 2010). Under physiological conditions, tau regulates the assembly and maintenance of the structural stability of microtubules (Barbier et al., 2019). In the diseased brain, however, tau becomes abnormally hyperphosphorylated, which ultimately causes the microtubules to disassemble, and the free tau molecules aggregate into paired helical filaments (Šimić et al., 2016). RV treatment inhibits tau aggregation and tau-induced toxicity in neuroblastoma cells and reduces the levels of phosphorylated tau and synapse loss in the brain of tau transgenic mice (Sun et al., 2019). Yu et al. (2018), using transgenic mice that overexpress human tau, demonstrated that RV was able to reduce the level of total hyperphosphorylated tau. According to the authors, these results can be related to the interruption of late-stage tau aggregation. Moreover, studies have shown that RV induces the activity of phosphoseryl/phosphothreonylproteinphosphatase-2A (PP2A), a highly complex heterotrimeric enzyme that has an important role in the homeostasis of tau, reducing its phosphorylation (He et al., 2017; Schweiger et al., 2017). Thus, these data indicate that RV could be an important botanical compound acting against Tau phosphorylation.

Some literature data have reported that RV can interfere with Aβ production, clearance, and aggregation, which is linked to microglial activation, neuroinflammation, apoptosis and neuronal death in AD (Jia et al., 2017). In an investigation using a transgenic model of AD in a *C. elegans* strain, RV reduces the Aβ-induced toxicity by acting on proteins involved in proteostasis and reducing the amount of Aβ aggregates (Regitz et al., 2016). Reinforcing the effects of RV on the clearance of Aβ, Vingtdeux et al. (2010) showed that RV activates AMP-activated protein kinase (AMPK) in neuronal cells and consequently reduces the Aβ levels and deposition, indicating that AMPK signaling mediates the amyloidogenic effect of RV.

Cholesterol metabolism is another mechanism that has been implicated in the pathophysiology of AD. It has been shown that patients carrying the ε4 allele of the apolipoprotein E (APOE4) are at a higher risk of developing AD, and it has been suggested that APOE4 could be a target for the treatment of this disease (Uddin et al., 2019). In this context, it has been shown that RV may regulate cholesterol metabolism and/or APOE4 expression in different experimental models (Thomas et al., 2014), however, the effects of RV in APOE4 expression and cholesterol metabolism are not known in the context of AD and should be better explored by further studies.

Thus, these data postulate that resveratrol is an important natural compound that could inhibit crucial processes involved in AD pathophysiology.

#### Neuroprotective Effects of Resveratrol in Parkinson’s Disease

In addition to the studies showing that RV could be neuroprotective against AD, several authors have investigated its effects in experimental models of PD. Several substances are known to produce damage in dopaminergic neurons and have been used in experimental models of PD, including 6-OHDA (6-hydroxydopamine), MPTP (1-methyl-4-phenyl-1,2,3,6-tetrahydropyridine) and rotenone. In this context, *in vitro* studies have shown that RV is neuroprotective against the damage produced by 6-OHDA (Albani et al., 2009; Zhang et al., 2015), MPP^+^ (a metabolite from MPTP; Gélinas and Martinoli, 2002; Alvira et al., 2007; Okawara et al., 2007; Bournival et al., 2009; Zeng et al., 2017) and rotenone (Lin et al., 2014, 2018; Feng et al., 2015; Wang et al., 2018a). These results are supported by studies in rodents showing that RV is neuroprotective against 6-OHDA (Jin et al., 2008; Huang et al., 2019), MPTP (Lu et al., 2008; Anandhan et al., 2010; da Rocha Lindner et al., 2015; Guo et al., 2016) and rotenone (Gaballah et al., 2016; Zhao et al., 2017; Palle and Neerati, 2018) counteracting the motor and cognitive changes induced by these neurotoxins as evidenced by several of these studies.

Several studies have associated the damage induced by these neurotoxins with the activation of apoptotic pathways. It has been shown that RV activates PI3K/Akt, a pro-survival signaling pathway, increases the ratio Bcl-2/Bax and decreases cytochrome C release and caspase-3 activation, therefore reducing apoptosis (Bournival et al., 2009; Zeng et al., 2017; Huang et al., 2019). Furthermore, several studies have associated the neuroprotective effects of RV with its antioxidant properties. In this context, studies have shown that RV decreases ROS production and/or increases antioxidant defenses after the exposure to MPTP/MPP^+^ (Okawara et al., 2007; Lu et al., 2008; Anandhan et al., 2010; da Rocha Lindner et al., 2015; Abolaji et al., 2018) and rotenone (Gaballah et al., 2016; Lin et al., 2018; Palle and Neerati, 2018). These results are supported by other studies showing that RV protects against mitochondrial dysfunction induced by experimental PD, counteracting the changes in mitochondrial morphology and mitochondrial membrane potential (ΔΨm; Zeng et al., 2017; Lin et al., 2018), as well as increasing mitochondrial biogenesis (Peng et al., 2016) and complex-I activity (Palle and Neerati, 2018).

As previously mentioned, RV is well known for being an activator of SIRT-1. In addition to the activation of other signaling pathways such as PI3K/Akt and the inhibition of caspase-3, studies have also shown that the neuroprotective effects of RV against rotenone may involve the activation of SIRT-1 and the suppression of p53 gene transcription, which is related to the up-regulation of pro-apoptotic proteins and cell death (Feng et al., 2015; Wang et al., 2018a). It should be mentioned, however, that other studies did not observe any involvement of SIRT-1 in the neuroprotective actions of RV against MPP^+^ (Alvira et al., 2007; Okawara et al., 2007).

Alpha-synuclein (α-syn) is an aggregate prone protein that has been closely associated with PD pathophysiology, contributing to mitochondrial dysfunction, neuroinflammation and neuronal death (Rocha et al., 2018). In addition to its neuroprotective effects in experimental models of PD induced by neurotoxins such as 6-OHDA, MPTP and rotenone, it has been shown that RV is also protective against the cell death triggered by α-syn. The neuroprotective effect of RV is related to decreased levels of ROS after α-syn exposure and seems to also involve the activation of SIRT-1 since it was blocked after the addition of sirtinol, a SIRT-1 inhibitor (Albani et al., 2009). The idea that α-syn may be at least partially involved in the neuroprotective effects of RV has been also supported by other studies showing that RV may stimulate the autophagic degradation of α-syn after SIRT-1 activation (Guo et al., 2016), as well as decrease α-syn expression in the striatum of mice after MPTP administration (Liu et al., 2019).

Autophagy is an important intracellular mechanism for protein degradation and the recycling of damaged proteins that avoids the accumulation of misfolded proteins that have been also involved in the pathophysiology of neurodegenerative diseases (Ghavami et al., 2014). Studies have shown that experimental parkinsonism is associated with autophagic dysfunction and that RV exerts neuroprotective effects by increasing the autophagic flux through the activation of HO-1 (Lin et al., 2014) and mitogen-activated protein kinase (MAPK) pathway (Lin et al., 2018).

The neuroprotective effects of RV have been also related to its anti-inflammatory activity and the regulation of astroglial activation. Jin et al. (2008) have shown that RV can reverse the increased expression of cyclooxygenase-2 (COX-2) and TNF-α in the substantia nigra of rats exposed to 6-OHDA. The involvement of glial cells in the protective actions of RV against experimental PD has been also explored. In this sense, Chang et al. (2013) have shown that RV decreases the expression of myeloperoxidase in glial cells, an enzyme that can oxidize NO and inhibit NO-dependent signaling in inflammation sites, and protected dopaminergic cells against rotenone. Interestingly, the neuroprotective effects of RV were observed only in neuron-glia co-cultures, while no protection was observed when RV was administrated in neurons cultured alone, pointing to an involvement of glial cells in the neuroprotective actions of RV. In agreement with these findings, Liu et al. (2019) showed that RV attenuates astroglial activation in the nigrostriatal pathway of mice exposed to MPTP. It is interesting to note that the authors showed that RV could present synergic effects when administrated in association with the dopamine precursor L-DOPA, which is still the major treatment available for PD patients. This is important since the administration of RV could allow the use of lower doses of L-DOPA, therefore decreasing its adverse effects, which are one of the major problems in its clinical use. Finally, it is important to mention that some studies have found that RV presented better efficacy when administered in nanoparticles when compared with the administration of free RV (da Rocha Lindner et al., 2015; Palle and Neerati, 2018), and further studies should better explore the effects of RV nanoparticles against neurodegenerative diseases.

### Clinical Studies and Limitations in the Use of Resveratrol

The preclinical evidence previously described supports the idea that RV is a promising compound for the prevention and treatment of neurodegenerative diseases, including AD and PD. Thus, some research groups have conducted clinical studies to evaluate whether RV is as effective in humans as shown in animal models of neurodegenerative diseases. These studies are summarized in Supplementary Table S1.

One of the first studies that evaluated the effects of RV on AD patients was conducted by Turner et al. (2015). This phase 2 clinical trial (randomized, placebo-controlled, double-blinded) was conducted in 119 individuals with mild to moderate AD that received RV 500 mg orally once daily (with dose escalation ending with 1,000 mg twice daily) or placebo for 52 weeks. The results evidenced that RV is safe and well-tolerated by the patients, however, no effects were observed on biomarkers of AD (plasma levels of Aβ 40 and 42; cerebrospinal fluid (CSF) levels of Aβ 40 and 42, tau and phosphorylated tau; hippocampal volume and entorhinal cortex thickness). Although this study did not show very promising results, the same research group analyzed samples of CSF and plasma from a subset of AD subjects with CSF Aβ 42 <600 ng/ml. In this small group of patients, they observed that RV decreases metalloproteinase (MMP) 9 in CSF, modulates neuroinflammation, induces adaptive immunity and attenuates the decline in cognition (Moussa et al., 2017). These results highlight the promising effect of RV in the modulation of neuroinflammation since matrix MMPs are a family of enzymes that degrade components of the extracellular matrix, which is important for normal blood-brain barrier function.

In another randomized placebo-controlled study, a small number of mild to moderate AD patients received a mixture of glucose, malate and a low dose of RV (5 mg/day), or placebo for 1 year. After treatment, it was possible to observe less deterioration (measured by specific scales to evaluate AD progress) in patients that received RV, however, these changes were not statistically significant (Zhu et al., 2018). Recently, a double-blind randomized controlled trial with 60 elderly individuals that received 200 mg/day of RV or placebo for 26 weeks failed to show significant benefits of RV on verbal memory performance (Huhn et al., 2018). Until the moment, no clinical trials are evaluating the effects of RV in PD.

The effects of RV intake were also investigated in several studies performed in humans with different health status. Most studies conclude that RV is safe in doses up to 5 g per day, however, some patients may have adverse gastrointestinal effects (Ramírez-Garza et al., 2018). It is important to highlight that studies have questioned the therapeutic application of RV due to its low bioavailability. The poor water solubility, the extensive first-pass effect, and phase II metabolism, due to its hydroxyl groups that are a substrate for conjugation reactions, are some of the reasons for RV reduced bioavailability (Intagliata et al., 2019). In this context, many researchers have been working on the design and synthesis of RV derivatives (RVD) with greater bioavailability (but similar pharmacological properties) than RV. These compounds are very promising since they could present better neuroprotective effects than RV *in vivo* and will be discussed in the next section.

## Resveratrol Derivatives (RVD)

**Figure 2:**
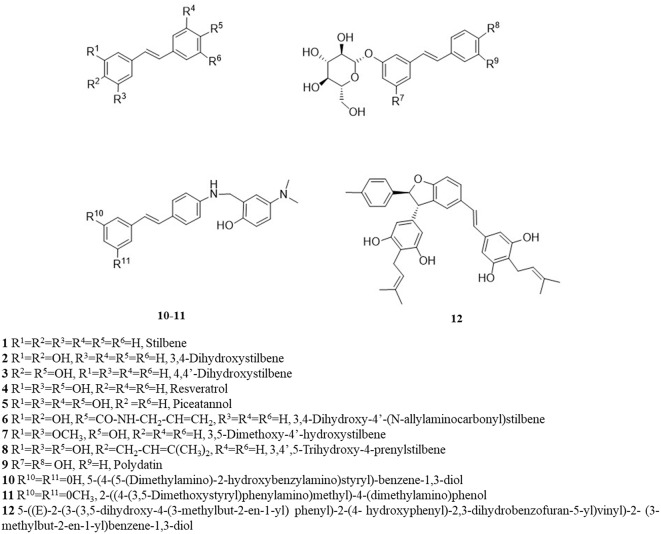
Chemical structure of resveratrol and its analogs.

RV is a small molecule (228 Da), with a quite simple chemical structure and equipped with several functional groups, that includes hydroxyphenyl and benzene diol groups, aromatic rings and a double-bound as a linker. The presence of these functional groups makes RV an attractive molecule to be modified into more effective derivatives (Lee et al., 2010; Li et al., 2016). Numerous synthetic methods can be used to modify the molecular structure of RV and the most common structural modifications founded in the literature are: hydroxylation; amination/amidation/imination; methoxylation; prenylation; and glycosylation (Figure 2). These structural modifications can lead to RVD with improved bioavailability and efficacy. Therefore, in this section, we present and discuss some studies involving the evaluation of the neuroprotective effects of RVD in experimental models of neurodegenerative diseases. After a broad search in the literature, the data that were found were divided into subgroups depending on the chemical structure of the derivatives. The main studies are briefly described in Supplementary Table S2.

### Hydroxylated Resveratrol Derivatives

Increasing the number of hydroxyl groups on the RV phenyl rings is an interesting strategy to enhance its water solubility and pharmacological activity (Saiko et al., 2008; Latruffe and Vervandier-Fasseur, 2018). In general, polyhydroxylated derivatives containing less than three hydroxyl groups in the stilbene portion exhibit very low oral bioavailability. This was evidenced by Chen W. et al. (2015) that show that the RVD trans-4,4′-dihydroxystilbene is slowly absorbed by the oral pathway and has low bioavailability in rats. However, compounds with four hydroxyl groups, such as oxyresveratrol and piceatannol (PCA; 3,3′,4,5′-trans-tetrahydroxystilbene) show a better water solubility, faster absorption and higher bioavailability than RV (Setoguchi et al., 2014; Chen et al., 2016). Setoguchi et al. (2014) showed that PCA is absorbed at levels that were 2-fold higher than RV in rats after intragastric administration. This study also indicates that PCA presents a higher metabolic stability than RV that predicts the best biological activity for PCA.

Following their influence in the pharmacokinetics, it has been shown that the number and position of additional hydroxyl groups play a crucial role in the antioxidant and anti-inflammatory effects of RV (Foti Cuzzola et al., 2012). For example, Cai et al. (2003) showed that RV analogs, 3,4-dihydroxytrans-stilbene and 4,4-dihydroxytrans-stilbene, are more effective antioxidants than RV. In the same way, five hydroxylated resveratrol analogs exerted a 6,600-fold higher free radical scavenging activity when compared to RV (Murias et al., 2005).

One of the most studied hydroxylated RVD is the PCA. Zhang et al. (2018) evaluated the effects of this compound on D-galactose-induced aging in mice. The results showed that PCA treatment retained spontaneous motor activity and improved spatial learning and memory skills, as well as prevented neuronal loss, reduced oxidative stress and promoted cell proliferation in the hippocampus and cortex. In hippocampal neuronal cells, PCA, similar to RV, protected against glutamate excitotoxicity by inducing HO-1 expression through Nrf-2 activation (Son et al., 2013).

Some studies evaluated the effects of PCA *in vitro* experimental models of AD. PCA treatment reduced intracellular accumulation of ROS and apoptosis induced by Aβ in the PC12 cell line. The protective effects of PCA against neuronal death were stronger than RV (Kim et al., 2007). PCA also displayed anti-amyloidogenic activity (inhibition of Aβ fibrils formation) and protected cultured hippocampal neurons against Aβ 25–35 and Aβ 1–42 toxicity, by a mechanism dependent on protein kinase C (Bastianetto et al., 2009). On the other hand, in experimental models of PD, PCA increased cell viability of G20119S-expressing neuronal cells (a leucine-rich repeat kinase-2-linked Parkinson’s disease model) and decreased the dopaminergic neurodegeneration, oxidative stress and locomotor deficits in a *Drosophila* (Angeles et al., 2016).

### Aminated, Amidated and Iminated Resveratrol Derivatives

Other structural modifications to the RV molecule include the addition of amine groups and the synthesis of amide and imine derivatives. Lu et al. (2013) synthesized some amino RVD and evaluated their effects as anti-AD agents. The derivatives 2-((4-(3,5-Dimethoxystyryl)phenylamino)methyl)-4-(dimethylamino)phenol (5 days) and (E)-5-(4-(5-(Dimethylamino)-2-hydroxy benzyl amino)styryl)-benzene-1,3-diol (10 days) were the lead compounds for AD treatment because they significantly inhibited Aβ aggregation, presented antioxidant activity and moderate acetylcholinesterase inhibition. Moreover, the 5 days compound can cross the blood-brain barrier *in vitro* and has low toxicity in mice.

In the work of Li et al. (2014), 20 imine RVD were designed as multitarget compounds for AD treatment. Most of the molecules inhibited Aβ aggregation, acted as antioxidants and metal chelators, and were neuroprotective against hydrogen peroxide in neuroblastoma cells. The compound 4-(((2-Hydroxyphenyl)imino)methyl)benzene-1,2-diol was the most promising agent, being more effective than RV as a neuroprotectant.

RV amidation has also been shown as a promising strategy for increasing antioxidant and neuroprotection capacity. According to Jung et al. (2009), amide derivatives are 2–3 times more potent as antioxidants than RV and are capable to inhibit LPS-induced NO generation in microglial cells. The allylamine analog trans-3,4-Dihydroxy-40-(N-allylaminocarbonyl)stilbene showed the best protective effect against glutamate excitotoxicity in primary cortical neuron cells (Jung et al., 2009).

### Methoxylated Resveratrol Derivatives

The methoxylation reaction is based on the addition of a methoxy group to a target chemical structure. Methoxylation can increase the metabolic stability of the molecule and the time until the peak plasma concentration is reached (Chimento et al., 2019). The most explored resveratrol-derived methoxylated molecule for neuroprotection is pterostilbene also called 3,5-Dimethoxy-4′-hydroxystilbene or 4-[(1E)-2-(3,5-Dimethoxyphenyl)ethenyl]phenol, an RV analog that substitutes two of the three hydroxyl groups with methoxy groups (Cichocki et al., 2008; Lin et al., 2009; Lange and Li, 2018). This main structural change increases the lipophilicity and enhances oral absorption and cellular uptake of pterostilbene in comparison with RV (Lin et al., 2009). Besides, pterostilbene is considered a less suitable substrate to human sulfotransferases when compared to RV, which can increase its metabolic stability and consequently its bioavailability (Wang and Sang, 2018).

Studies have proven that pterostilbene presents an improved pharmacokinetic profile when compared to RV. Hougee et al. (2005) compared the half-life of pterostilbene and RV in mouse plasma after intravenous administration, being 77.9 and 10.2 min, respectively. Another study demonstrated that animals exposed to different doses and periods showed that the bioavailability of pterostilbene was approximately 4-fold greater than of RV (Kapetanovic et al., 2011). Another methoxylated synthetic derivative of RV is the trans-3,4,5,40-tetra methoxy stilbene (DMU 212 or TMS), which demonstrated more favorable pharmacokinetic properties when compared to RV. Higher levels of the compound have been found in the brain, colon mucosae, and small intestine compared to RV (Sale et al., 2004).

Pterostilbene protects human SH-SY5Y neuroblastoma cells from H_2_O_2_ induced oxidative damage by restoring signaling pathways involved in cell growth and proliferation (PI3K/Akt and MAPK/ERK; Song et al., 2015). The authors also demonstrated that estrogen receptor α (ER-α) is involved in the neuroprotective actions of pterostilbene. Using the same cell line, other authors showed that pterostilbene protects against oxidative stress and mitochondrial dysfunction caused by hyperglycemia, through a mechanism involving the activation of Nrf2 signaling and its translocation to the nucleus (Yang et al., 2017).

In a model of Aβ (Aβ1–42)-induced neuroinflammation in BV-2 murine microglial cells, pterostilbene decreases the expression and levels of NO and proinflammatory cytokines (IL-6, IL-1β, and TNF-α). Furthermore, pterostilbene was able to inhibit the inflammasome NLRP3/caspase-1 (CASP1) pathway, being a promising agent for the treatment of AD (Li et al., 2018). Pterostilbene was more effective than RV in neuromodulating the deleterious effects of aging and AD. SAMP8 mice, animals widely used to study sporadic and age-related AD, were fed for 8 weeks with 120 mg/kg of pterostilbene and RV, equivalent to two glasses of wine. Pterostilbene presented better results than RV and improved the cognitive function of animals in the radial arm water maze test. Upregulation of peroxisome proliferator-activated receptor (PPAR) alpha expression could be associated with modulation of markers of cellular stress, inflammation, and AD pathology, such as the expression manganese superoxide dismutase (MnSOD), transcription of NF-κB and JNK phosphorylation, all significantly improved by pterostilbene. Also, high levels of pterostilbene in serum and brain samples were found in this study, while RV was detected in low levels in the serum and was undetectable in the brain (Chang et al., 2012). In the study of Joseph et al. (2008), 19-month-old Fischer 344 rats were fed with a low (2.5 mg/kg) or a high (10 mg/kg) dose of pterostilbene for 12–13 weeks. Both diets with pterostilbene were able to reverse the deleterious effects of aging on the cognitive function, especially the working memory, in a dose-dependent manner. The best performance of pterostilbene can be attributed to its increased lipophilicity in comparison to RV, as a result of the replacement of the hydroxyl group of RV by a methoxy group in pterostilbene (Cichocki et al., 2008). Such alteration may increase the bioavailability of pterostilbene, making its neuroprotective effects more potent. Interestingly, Hou et al. (2014) have also shown that pterostilbene presents anti-inflammatory effects and improves the cognitive function of mice exposed to a model of neuroinflammation, a key component in the pathophysiology of neurodegenerative diseases, represented by the bilateral intrahippocampal injection of LPS.

Some studies have demonstrated the low toxicity of pterostilbene in animal models and humans. Ruiz et al. (2009) evaluated the possible harmful effects of ingesting high doses of pterostilbene in Swiss mice for a subchronic period. Animals received pterostilbene at doses of 0, 30, 300, and 3,000 mg/kg body weight/day for 28 days. The authors observed no mortality, no differences in food or water consumption, neither changes in histopathological or biochemical parameters of the animals. On the other hand, a double-blind placebo-controlled intervention clinical trial evaluated the safety of long-term pterostilbene administration in humans. The patients with hypercholesterolemia received pterostilbene at doses of 0, 50, and 125 mg twice a day. At the end of 8 weeks, it was not observed adverse drug reactions on glucose, renal, and hepatic markers. Thus, the authors report that doses of pterostilbene up to 250 mg/day are safe for humans (Riche et al., 2013).

### Prenylated Resveratrol Derivatives

Prenylation is the covalent binding reaction of a hydrophobic/lipid moiety to a molecule of interest. Literature data indicate that this chemical modification increases the bioactivity of the backbone compound with no prenylation (Chen et al., 2014). Prenylated RV derivatives have also shown promising results for the development of drugs for AD (E)-3,5,40-Trihydroxy-4-prenylstilbene showed free radical scavenging, good anti-Aβ aggregation and moderate inhibition of β-secretase (BACE1), further neuroprotective and neuritogenic activities. The neuroprotective effect was superior to that presented by RV and was even similar to the positive control with quercetin. The replacement of the -OH groups of RV structure is associated with promising activity presented by the compound against AD (Puksasook et al., 2017).

The enzyme monoamine oxidase-B (MAO-B) acts on dopamine degradation when it is recaptured from the extracellular medium to the cytosol (Thorpe et al., 1987). MAO-B may also be associated with abnormal production of γ-aminobutyric acid in reactive astrocytes, thus emerging as a potential therapeutic target of AD (Park et al., 2019). Two RV isoprenylation dimer derivatives showed moderate inhibition of human MAO-B activity. Both compounds also showed high antioxidant capacity in FRAP, DPPH and ABTS assays. Through *in vitro* assays, both derivatives demonstrated low toxicity and neuroprotective activity in PC12 cells exposed to oxidative molecules such as H_2_O_2_, rotenone, and oligomycin-A, and in BV2 microglial cells exposed to inflammatory stimulation with LPS. In addition, the compound 5-((E)-2-(3-(3,5-dihydroxy-4-(3-methylbut-2-en-1-yl)phenyl)-2-(4-hydroxyphenyl)-2, 3-dihydrobenzofuran-5-yl)vinyl)-2-(3-methylbut-2-en-1-yl)benzene-1,3-diol presents increased capacity to transpose the blood-brain barrier, demonstrating the importance of the alkyl group (Tang et al., 2019).

### Glycosylated Resveratrol Derivatives

The glycosylation reaction is characterized by the addition of one (or more) glycosidic moieties to the molecule. This process can increase the water solubility and stability of RV. The RVD glycosylated molecule most exploited for neuroprotection is polydatin (3,4′,5-Trihydroxystilbene-3-O-β-D-glucopyranoside), also known as piceid. Polydatin has the same biological properties as RV, but presents greater bioavailability and is less susceptible to enzymatic oxidation.

Zhou et al. (2009) investigated the absorption and metabolism of polydatin in rats after oral administration by gavage at three different doses (50, 100, and 300 mg/kg). They observed a dose-dependent absorption and a nonlinearly dose-dependent metabolism of polydatin in rats. Ding et al. (2014) analyzed the pharmacokinetics and bioavailability of polydatin in rats after intravenous and oral administration. The pharmacokinetic parameters demonstrated dose-dependent behavior regardless of the route of administration, dose increase reflected an increase in the terminal half-life of the polydatin. However, the higher increase in peak plasma concentrations and more rapid elimination was observed in the intravenous administration when compared to the oral administration. A comparative study between polydatin and RV observed that when they were administrated at the same doses, the serum concentration of polydatin was 3–4-fold higher than RV. Furthermore, other authors have shown that polydatin may present a higher oral absorption than RV (Wang H. L. et al., 2015). A study using polydatin for the treatment of urate nephropathy in mice revealed that at doses up to 50 mg/kg, polydatin presented no side-effects in the liver, kidney, and immune system (Chen et al., 2013).

As well as RV, polydatin has antioxidant and anti-inflammatory properties (Fabris et al., 2008; Lanzilli et al., 2012). Chen Y. et al. (2015) investigated the effects of polydatin in three classical rodent models of PD, induced by rotenone, MPTP and 6-OHDA. The treatment with polydatin prevented motor impairments and changes in oxidative stress markers (GSH, thioredoxin, MDA, and SOD). Moreover, the rotenone-induced dopaminergic neurodegeneration was decreased when the animals received polydatin. In order to elucidate some of the mechanisms involved in neuroprotection exhibited by polydatin, one study used human SH-SY5Y neuroblastoma cells exposed to oxidative stress induced by dopamine. Polydatin was able to protect the cells against oxidative damage and such neuroprotection could be associated with the activation of the MAP kinase pathway (ERK1/2 and ERK5), since the inhibition of this pathway resulted in a decreased protective effect of polydatin. The activation of the dopamine-induced caspase pathway was also inhibited by polydatin pretreatment (Potdar et al., 2018). In a rat model of LPS-induced PD, polydatin ameliorated motor dysfunction and protected dopaminergic neurons through the decrease of microglial activation and pro-inflammatory mediators. Furthermore, the levels of p-Akt and Nrf-2 were increased and the activation of NF-κB was suppressed by polydatin in the substantia nigra (Huang et al., 2018).

Studies on the effects of glycosylated RVD in AD models are slightly scarcer. In an *in vitro* model of AD, polydatin, as well as RV, showed dose-dependent inhibition on Aβ 25–35 polymerization, which is one of the major characteristics of AD (Rivière et al., 2007). Therefore, polydatin and other glycosylated forms of RV may be promising compounds for the prevention and treatment of age-associated deficits and neurodegenerative diseases such as AD and PD.

## Conclusion and Future Directions

In summary, several pieces of evidence have shown in the last decades that RV presents neuroprotective actions in experimental models of AD and PD. However, clinical trials have failed to demonstrate these actions, probably due to the low bioavailability of RV, among other pharmacokinetics characteristics. Therefore, studies have focused on the development of RVD, which could retain the neuroprotective properties of RV, but presenting higher bioavailability and efficacy. In this context, different RVD has been generated, including hydroxylated, aminated, amidated, iminated, methoxylated, prenylated and glycosylated derivatives. These derivatives are neuroprotective in some experimental models of AD and/or PD, however, more preclinical studies are needed to understand their mechanisms of action and toxicity before testing them in clinical trials. It is important to mention that several authors have coincided in showing that RVD may be more effective neuroprotectants in preclinical studies than RV, probably due to their improved pharmacokinetics. Therefore, RVD could be promising agents for the development of new treatments against neurodegenerative diseases. The discovery of new molecules for the treatment of these diseases could have a great impact worldwide, not only improving the outcomes of patients but also potentially decreasing the amount of money that is currently spent in their treatment. It should be mentioned, however, that although some studies have shown the influence of sex in the pathophysiology of neurodegenerative diseases (as reviewed by Ullah et al., 2019), the vast majority of *in vivo* studies cited in this review were performed in males. Therefore, future studies should also evaluate the effect of RV and its derivatives in female animals, aiming to identify if these compounds could be effective in both sexes, or if they present sex-specific effects.

## Author Contributions

BA, RN-N, LS, MS, DC-S, and MH wrote the manuscript and designed the figures and tables. CA-M and AM-B helped to write the article. All authors reviewed and approved the final version of the manuscript.

## Conflict of Interest

The authors declare that the research was conducted in the absence of any commercial or financial relationships that could be construed as a potential conflict of interest.
