# Supplementary material for: Resveratrol Derivatives as Potential Treatments for Alzheimer’s and Parkinson’s Disease
Source: Front Aging Neurosci. 2020 Apr 17;12:103. doi: 10.3389/fnagi.2020.00103 (PMC7180342; doi:10.3389/fnagi.2020.00103)
Supplement: Supplementary file 1 [file Table_1.DOCX]

Table 1 – Clinical trials evaluating the effects of resveratrol in neurodegenerative diseases.

| **Type of study** | **Sample** | **Dose** | **Duration** | **Main results** | **Reference** |
| --- | --- | --- | --- | --- | --- |
| Double-blind randomized controlled trial (phase 2) | 119 patients with mild to moderate AD (n=64 treated with RV and n=55 treated with placebo) | RV 500 mg orally OD with dose escalation: 500 mg every 13 weeks (until 1,000 mg BID) | 52 weeks | Safe and well tolerated: nausea, diarrhea, and weight loss were the most frequent adverse effect.  No benefit was observed on biomarkers assessed: plasma and CSF Aβ_40_ and Aβ_42_, CSF tau protein, hippocampal volume and entorhinal cortex thickness. Furthermore, RV led to increased brain volume loss. | (Turner et al. 2015) |
| Retrospective study (using samples from the study by Turner et al., 2015) | Patients with CSF Aβ_42_ <600 ng/ml (n= 19 treated with RV and n= 19 treated with placebo) | RV 500 mg orally OD with dose escalation (maximum dose 1,000 mg BID) | 52 weeks | RV-treated *vs.* placebo: ↓↓ CSF MMP9, ↑ MDC, ↑ IL-4 and ↑ FGF-2. | (Moussa et al., 2017) |
| Double-blind randomized controlled trial (phase 3) | 32 patients with mild to moderate AD (n=17 intervention group and n=15 placebo group) | RV (5 mg/day) in a mixture of glucose and malate (15ml) | 52 weeks | Safe and well tolerated (low-dose).  No significant changes on Alzheimer’s Disease Assessment Scale–cognitive subscale, Mini–Mental State Examination, Alzheimer’s Disease Cooperative Study–Activities of Daily Living Scale or Neuropsychiatric Inventory. | (Zhu et al., 2018) |
| Double-blind randomized controlled trial | 60 healthy elderly individuals (n=30 intervention group and n=30 placebo group) | RV 200 mg OD | 26 weeks | No significant benefits of RV on verbal memory performance. | (Huhn et al., 2018) |

**Symbols and abbreviations:** ↓: decrease; ↑: increase; BID: twice daily; CSF: cerebrospinal fluid; FGF: fibroblast growth factor; IL: interleukin; MDC: macrophage-derived chemokine; MMP: metalloproteinase; OD: once daily; RV: resveratrol.
