# Supplementary material for: Resveratrol Derivatives as Potential Treatments for Alzheimer’s and Parkinson’s Disease
Source: Front Aging Neurosci. 2020 Apr 17;12:103. doi: 10.3389/fnagi.2020.00103 (PMC7180342; doi:10.3389/fnagi.2020.00103)
Supplement: Supplementary file 2 [file Table_2.DOCX]

Table 2: Effects of resveratrol derivatives in neurodegenerative disease models.

| **Modification** | **Derivative** | **Experimental model** | **Dose or concentration** | **Main Effects** | **Reference** |
| --- | --- | --- | --- | --- | --- |
| Hydroxylation | Piceatannol (Trans-3,3',4,5'-trihydroxystilbene) | PC12 cells exposed to Aβ | 20 µM | Reduction of intracellular ROS production and apoptosis | Kim et al. 2007 |
|  |  | Primary hippocampal (neuronal/glial) cells exposed to Aβ | 25 µM | Protection against Aβ-induced toxicity | Bastianetto et al., 2009 |
|  |  | HT22 neuronal cells exposed to glutamate | 10 µM | Protection against glutamate-induced oxidative stress by activation of Nrf-2 | Son et al., 2013 |
|  |  | Leucine-rich repeat kinase-2-linked Parkinson’s disease model in neuronal cells and Drosophila | 10 µmol/L | Reduction of dopaminergic neurons death, oxidative stress and locomotor deficits | Angeles et al., 2016 |
|  |  | D-galactose-induced mouse model of aging | 20 mg/kg | Prevention of behavioral disorder and neuronal death | Zhang et al., 2018 |
| Amination | 2-((4-(3,5-Dimethoxystyryl)phenylamino)methyl)-4-(dimethylamino)phenol (5d) and (E)-5-(4-(5-(Dimethylamino)-2-hydroxybenzylamino)styryl)-benzene-1,3-diol (10d) | Aβ aggregation *in vitro* | 7.56 µM and  6.51 µM,  respectively | Inhibition of Aβ aggregation | Lu et al., 2013 |
| Imination | 4-(((2-Hydroxyphenyl)imino)methyl)benzene-1,2-diol | Aβ aggregation *in vitro* and SH-SY5Y neuroblastoma cells exposed to H_2_O_2_ | 1.25 µM -10 µM | Inhibition of Aβ aggregation and neuroprotection | Li et al., 2014 |
| Amidation | trans-3,4-Dihydroxy-40-(N-allylaminocarbonyl)stilbene | Primary cortical neurons exposed to glutamate | 10 µM-50 µM | Neuroprotection | Jung et al., 2009 |
| Methoxylation | Pterostilbene | Old Fischer 344 rats | 2.5 and 10 mg/kg body weight | Improvement of cognitive deficit induced by aging | Joseph et al., 2008 |
|  |  | Mice model of sporadic and age-related AD (SAMP8) | 120 mg/kg of diet | Improvement of cognitive function, reduction of cellular stress and inflammatory markers | Chang et al., 2012 |
|  |  | SH-SY5Y neuroblastoma cells exposed to H_2_O_2_ | 1-10 nM | Neuroprotection | Song et al., 2015 |
|  |  | BV-2 microglial cells exposed to Aβ | 5 and 10 µM | Attenuation of neuroinflammation | Li et al., 2018 |
| Prenylation | (E)-3,5,40-Trihydroxy-4-prenylstilbene | Aβ aggregation and BACE1 activity *in vitro* assay | 4.78 and 50 µM, respectively | Inhibition of Aβ aggregation and of BACE1 | Puksasook et al., 2017 |
|  | 5-((E)-2-(3-(3,5-dihydroxy-4-(3-methylbut-2-en-1-yl)phenyl)-2-(4-  hydroxyphenyl)-2,3-dihydrobenzofuran-5-yl)vinyl)-2-(3-methylbut-2-en-1-yl)benzene-1,3-diol  5-((E)-2-(3-(5-hydroxy-2,2-dimethylchroman-7-yl)-2-(4-hydroxyphenyl)-2,3  -dihydrobenzofuran-5-yl)vinyl)-2-(3-methylbut-2-en-1-yl)benzene-1,3-diol | MAO-B *in vitro* activity assay and PC12 cells exposed to H_2_O_2_, rotenone, and oligomycin-A | 0.92 and 3.93 µM;  25 µM;  and 15 µM | Inhibition of MAO-B activity and neuroprotection against oxidative damage | Tang et al., 2019 |
| Glycosylation | Polydatin | Aβ polymerization *in vitro* assay | 4.7 µM | Prevention of Aβ aggregation | Rivière et al., 2007 |
|  |  | PD models induced by rotenone and 6-OHDA in rats and, by MPTP in mice. | 80 mg/kg;  50 mg/kg;  100 and 200 mg/kg, respectively. | Prevention of motor impairments and changes in oxidative stress markers, and reduction of dopaminergic neurodegeneration | Chen et al., 2015 |
|  |  | SH-SY5Y neuroblastoma cells exposed to dopamine | 30 µM | Protection against oxidative damage through the activation of the MAPK pathway | Potdar et al., 2018 |
|  |  | Rat model of PD induced by LPS | 25-400 µM | Amelioration of motor dysfunction and protection of dopaminergic neurons through the decrease of microglial activation and pro-inflammatory mediators | Huang et al., 2018 |

Abbreviations: 6-OHDA – 6-hydroxydopamine; Aβ – amyloid-beta; AD – Alzheimer’s Disease; BACE – beta-secretase; LPS – lipopolysaccharide; MAO – monoamine oxidase; MAPK – mitogen-activated protein kinase; MPTP – 1-methyl-4-phenyl-1,2,3,6-tetrahydropyridine; Nrf 2 – nuclear factor erythroid 2-related factor; PD – Parkinson’s Disease; ROS – reactive oxygen species.
